# Multi-Domain Robot Swarm for Industrial Mapping and Asset Monitoring: Technical Challenges and Solutions

**DOI:** 10.3390/s25206295

**Published:** 2025-10-11

**Authors:** Fethi Ouerdane, Ahmed Abubaker, Mubarak Badamasi Aremu, Mohammed Abdel-Nasser, Ahmed Eltayeb, Karim Asif Sattar, Abdulrahman Javaid, Ahmed Ibnouf, Sami El Ferik, Mustafa Alnasser

**Affiliations:** 1 Control & Instrumentation Engineering Department, King Fahd University of Petroleum and Minerals, Dhahran 31261, Saudi Arabia; 2 Interdisciplinary Research Center for Smart Mobility & Logistics, King Fahd University of Petroleum and Minerals, Dhahran 31261, Saudi Arabia; 3 Industrial & Systems Engineering, King Fahd University of Petroleum and Minerals, Dhahran 31261, Saudi Arabia; 4 Research & Development, Yokogawa Saudi Arabia Company, Dhahran 31952, Saudi Arabia

**Keywords:** UAV–UGV collaboration, SLAM, vision-based landing, ROS, Simulink, AprilTags, real-time communication, digital meter reading, YOLOv5, hardware-in-the-loop (HIL) testing

## Abstract

Industrial environments are complex, making the monitoring of gauge meters challenging. This is especially true in confined spaces, underground, or at high altitudes. These difficulties underscore the need for intelligent solutions in the inspection and monitoring of plant assets, such as gauge meters. In this study, we plan to integrate unmanned ground vehicles and unmanned aerial vehicles to address the challenge, but the integration of these heterogeneous systems introduces additional complexities in terms of coordination, interoperability, and communication. Our goal is to develop a multi-domain robotic swarm system for industrial mapping and asset monitoring. We created an experimental setup to simulate industrial inspection tasks, involving the integration of a TurtleBot 2 and a QDrone 2. The TurtleBot 2 utilizes simultaneous localization and mapping (SLAM) technology, along with a LiDAR sensor, for mapping and navigation purposes. The QDrone 2 captures high-resolution images of meter gauges. We evaluated the system’s performance in both simulation and real-world environments. The system achieved accurate mapping, high localization, and landing precision, with 84% accuracy in detecting meter gauges. It also reached 87.5% accuracy in reading gauge indicators using the paddle OCR algorithm. The system navigated complex environments effectively, showcasing the potential for real-time collaboration between ground and aerial robotic platforms.

## 1. Introduction

In recent years, robot-to-robot communication and heterogeneous multi-robot systems have gained significant attention across various domains, including industrial automation, surveillance, and environmental monitoring [1,2,3]. One prominent application is the collaboration between unmanned ground vehicles (UGVs) and unmanned aerial vehicles (UAVs) for indoor exploration, mapping, and inspection tasks. These systems combine the complementary strengths of each platform; for example, aerial drones offer high-resolution visual coverage and top-down perspectives, while ground robots provide stable platforms for extended operation and fine-resolution mapping using LiDAR and other proximity sensors. In complex indoor industrial environments, where GPS is unavailable and layouts are cluttered with static and dynamic obstacles, real-time mapping and localization are essential. Traditional ground robots equipped with LiDAR can generate accurate 2D maps of such environments, while UAVs can be deployed to inspect areas inaccessible to ground platforms.

Such hybrid systems also face multiple challenges associated with implementing a heterogeneous system comprising a ground-based vehicular robot and a drone for cooperative mapping of an indoor area [4,5,6]. This paper focuses on addressing the challenges associated with implementing the aforementioned heterogeneous systems to facilitate the development of an autonomous gauge inspection system, while simultaneously enabling the mapping of complex indoor environments. To support such coordination, the ground robot must be capable of reliable autonomous navigation using real-time mapping. This work implements a LiDAR-based SLAM system on the UGV to construct occupancy grid maps of the environment, ensuring accurate localization. These capabilities are crucial for obstacle avoidance, positioning inspection points, and enabling precise UAV deployment and recovery. The UAV component handles image acquisition and inspection, including vision-based landing on the UGV and digital meter gauge reading. A resilient communication framework enables coordination between agents, while computer vision algorithms are used to extract readings from meter images.

The purpose of this study is to develop a multi-robot system that automates industrial inspection tasks by combining the mapping and navigation capabilities of a UGV with the sensing and inspection capabilities of a UAV. This paper presents the system architecture, implementation, and experimental validation of this heterogeneous platform, addressing the technical challenges of indoor SLAM, UAV–UGV coordination, vision-based landing, and AI-driven meter reading.

### Main Contributions

The key contributions of this work are summarized as follows:

- A UAV–UGV collaborative framework for industrial inspection and asset monitoring, supporting real-time coordination between aerial and ground robots via event-triggered TCP/IP communication in ROS and Simulink environments.
- Implementation of a LiDAR-based SLAM system on a UGV (TurtleBot 2) for real-time map generation and autonomous navigation in cluttered indoor environments. The SLAM Toolbox was fine-tuned for mapping accuracy and validated through RViz and Gazebo simulations.
- Design of a vision-based autonomous UAV landing system using AprilTags, with pose estimation supported by OptiTrack ground-truth validation. The system combines visual tracking with onboard Proportional-Integral-Derivative (PID) and Proportional-Integral-Velocity (PIV) control loops to enable landing on a mobile UGV platform.
- Development of an end-to-end digital meter gauge reading pipeline that combines YOLOv5 for object detection, U-Net for region segmentation, and PaddleOCR for numerical value extraction, achieving 87.5% accuracy on real-world UAV-captured images.
- Establishment of a HIL simulation environment for validating swarm behavior, vision algorithms, and inspection routines under realistic constraints. The Quanser Quarc platform enabled direct deployment of Simulink models to UAV hardware.
- Experimental validation in both simulation and physical lab environments was conducted, demonstrating the system’s feasibility for multi-domain robotic collaboration under real-world conditions, including communication latency, sensor noise, and environmental variability.

**Figure 1:**
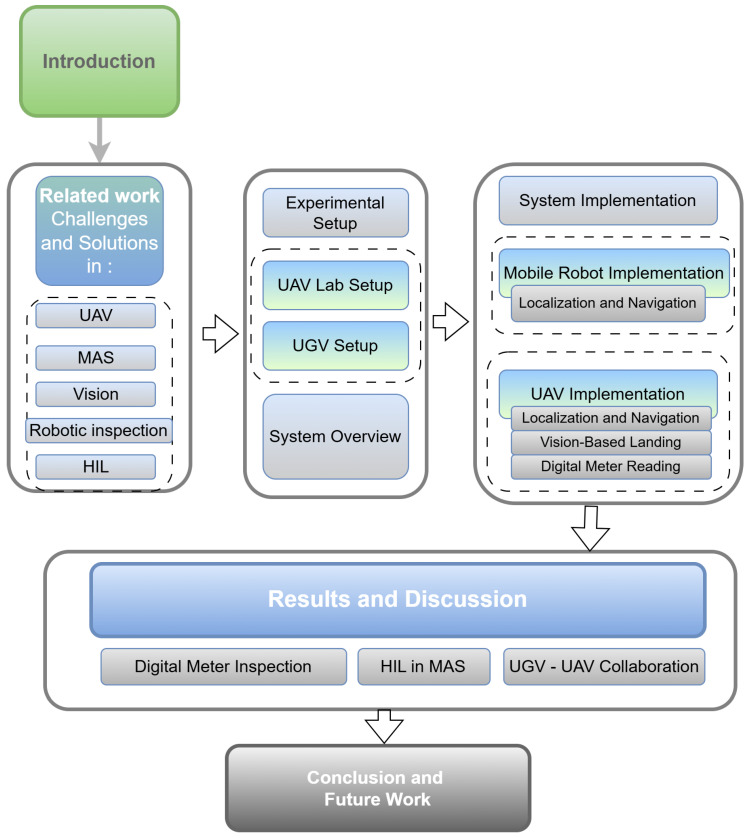
Overview of the paper’s organizational structure, illustrating the progression from related work and experimental setup through system implementation, results, and conclusions in the context of UAV–UGV collaborative inspection.

Figure 1 provides a visual overview of the paper’s structure, designed to help readers navigate the discussion of UAV and UGV systems for inspection tasks. The flow begins with an introduction that sets the stage by addressing the broader context and challenges of autonomous systems in industrial inspection. Following this, Section 2 presents a comprehensive review of related work, summarizing advancements in UAV technology, multi-agent coordination, vision-based systems, and multi-domain robotic swarms for industrial environments. Section 3 provides an overview of the proposed system, detailing its architecture and functional components. The experimental setup is described in Section 4, highlighting the integration of the UAV and UGV platforms to ensure clarity on how the systems are designed and tested. Section 5 focuses on the practical applications of the system, including localization, navigation, and vision-based landing techniques. The results and a discussion are presented in Section 6, synthesizing the findings and offering insights into system performance and operational considerations. Finally, Section 7 concludes the paper by summarizing the contributions and outlining potential future research directions. The figure effectively ties these sections together, presenting the flow of ideas and methodologies coherently.

## 2. Related Work

This section discusses the localization of robots, the interoperability of UAVs with land vehicles, and the mapping of the indoor environment. Multi-robot localization involves accurately and consistently coordinating the position and movement of multiple robots. This often requires integrating data from various sensors across the robots to overcome individual localization errors. Odometry error is a significant challenge in multi-robot localization. Accumulation of errors in the robot’s internal odometry can lead to significant discrepancies in the robot’s positions when operating in dynamic environments [7]. Effective handling of such uncertainty requires advanced techniques such as probabilistic models (e.g., Kalman filters, particle filters) or robust optimization approaches that ensure the system remains functional despite the presence of noise and errors. Furthermore, uncertainty in communication between robots adds another layer of complexity to synchronization and coordination tasks [7,8].

The integration of UAVs and UGVs adds another layer of complexity to a network topology. Aloqaily et al. [9] suggested deploying a swarm of UAVs to collaborate with connected land vehicles. This solution offers significant advantages in terms of reducing latency and enabling faster data delivery. However, as noted in [9], the dynamic nature of the environment, where both UAVs and land vehicles are in motion, requires the network to adapt in real time, recalculating the optimal route as conditions change. This is more difficult in indoor areas, especially industrial environments, where mobility patterns of both UAVs and land vehicles are less predictable. Since UAVs are battery-powered devices, their participation in the network can lead to the quick depletion of resources. A well-balanced strategy is necessary to prevent UAVs from depleting their energy too quickly, which would negatively impact the overall network performance.

The concept of leader-follower architectures emerges as a promising approach for coordinated multi-robot systems. This framework allows a UAV, equipped with advanced vision sensors and navigation capabilities, to act as a leader, providing real-time guidance and coordination to UGVs for tasks such as inspection, surveillance, and material handling within complex industrial environments.

The leader-follower approach is often preferred for its simplicity and low communication demands [10,11,12], making it highly effective across a range of applications. The authors in [13] proposed a novel leader-follower cooperation technique, focusing on enhancing the functionality and efficiency of such systems within the context of intelligent transportation, specifically exploring its application to UAV–UGV heterogeneous systems.

Heterogeneous robotic systems in Safety, Security, and Rescue Robotics (SSRR) are increasingly focusing on integrating advanced technologies, including sensors, communication protocols, GUIs, object detection, volume estimation, and 3D reconstruction to improve real-time data collection, hazard identification, and victim localization. Among the notable research in this field, the study in [14] introduced a novel approach that leveraged UAVs, UGVs, and USVs in collaborative SSRR scenarios. His research focused on optimizing path planning and decision-making for multi-robot systems (MRS), addressing critical challenges such as the limitations of conventional cooling methods in hazardous environments. To overcome these constraints, specialized electronic enclosures and cooling mechanisms have been proposed. By demonstrating how UAVs provide aerial guidance to ground and water robots navigating autonomously or semi-autonomously to targets, the study showcased practical applications and highlighted the adaptability of heterogeneous robotic systems in complex rescue operations. The increasing complexity of simulation and modeling poses a significant challenge in the development of heterogeneous, multi-agent, collaborative robotic systems [15,16]. Early simulation efforts relied heavily on modular tools integrated into established platforms, such as Simulink, which allowed researchers to customize simulations for specific robotic tasks. This was especially true with the introduction of diverse robotic agents possessing unique physical structures, sensor configurations, and operational roles. This diversity has presented challenges for traditional simulation approaches, as managing the intricate interactions within such heterogeneous systems requires more sophisticated simulation techniques.

To address these challenges, HIL testing emerges as a crucial methodology for validating safety-critical systems. By integrating the actual embedded system within a simulated environment, HIL testing provides a realistic evaluation of system behavior under various conditions. This approach is particularly valuable in domains such as automotive and aerospace, where rigorous testing is essential [17]. In the context of our research, which involves UAVs and UGVs for meter gauge inspection, HIL testing offers a robust platform for evaluating the performance of the flight controller and its interaction with the simulated environment, ensuring the safety and reliability of the UAV system before deployment.

**Figure 2:**
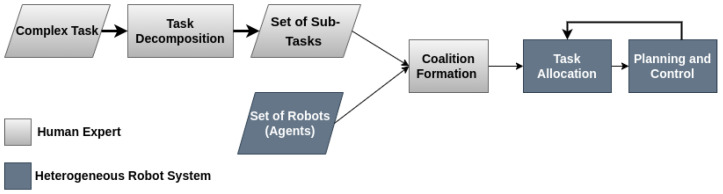
Cooperative Heterogeneous Multi-Robot Systems.

The study in [18] provides a comprehensive survey of cooperative heterogeneous MRS and their application in automating complex tasks. The authors first contextualized MRS within the broader field of multi-agent systems before delving into specific challenges and future research directions. They categorized tasks assigned to MRS by their complexity, noting that task complexity influenced both the number and type of robots required. Task complexity and coordination represent another critical aspect of MRS development [18]. Unlike the study in [15], the focus in [18] was not on the technical complexity of integrating diverse robotic agents through advanced simulation architectures, but shifted to how these systems handled varying task complexities in real-world scenarios. Their survey emphasized that task complexity directly impacts the number and type of robots needed, as well as the degree of coordination required for successful execution. Loosely coordinated tasks, such as large-scale mapping or hazardous material cleanup, can be divided into independent sub-tasks. In contrast, tightly coupled tasks, such as object transport or robot soccer, require high levels of interaction and synchronized execution. To address this, the authors proposed a systematic framework for MRS design, encompassing task decomposition, coalition formation, task allocation, and task execution. This structured approach complemented [15] and emphasized robust simulation, providing a comprehensive strategy for both the virtual modeling and real-world deployment of heterogeneous MRS in complex environments, as shown in Figure 2.

As heterogeneous MRS collaborate to address complex tasks, the integration of advanced sensor technologies becomes crucial, especially for ensuring accurate localization and effective task execution. Within this context, SLAM is the foundational methodology for enabling robots to navigate and map unknown environments. The continued evolution of SLAM, driven by diverse sensor technologies, has significantly improved the accuracy and reliability of mapping processes in various robotic applications. For instance, different SLAM-based mapping technologies tailored to indoor environments, emphasizing the role of advanced sensor setups like LiDAR and depth cameras, have been examined in [19]. These technologies are essential in achieving high levels of accuracy in challenging indoor settings, such as narrow corridors or complex structures like libraries. Furthermore, the research in [20] explored the integration of hardware configurations with the Google Cartographer SLAM algorithm, focusing on optimizing setups for tasks such as decontamination. The research advocates a multi-sensor approach, incorporating LiDARs, inertial measurement units, and odometry, to enhance accuracy in real-world simulations. These advancements in sensor integration and SLAM methodologies highlight the ongoing efforts to refine the mapping and localization capabilities of robot systems, making them more adaptable and effective in complex environments.

Choosing the right type of map remains crucial for successful multi-robot SLAM. For ground robots navigating indoors, a simple 2D map often does the trick. Research has shown that occupancy grids are a good balance between accuracy and efficiency compared to maps based on features [21]. For long-term operations, the choice of map representation becomes more crucial for applications involving UAV–UGV communication, as unnecessary data exchange could impact computational power and memory usage. As robots operate for extended periods, memory requirements inevitably increase, posing a significant challenge. Communication bottlenecks often arise from the excess exchange of sensor data for map representations [22].

In addition to the mentioned sensors, vision-based navigation has become a widely adopted approach in UAV technology [23,24,25,26,27]. Although other methods, such as those using LiDAR or GPS, provide essential means for navigation, vision-based navigation is particularly advantageous due to its low onboard hardware requirements, which reduce both the weight and cost of the UAV. This method utilizes onboard image processing to perceive the environment around the UAV, enabling it to navigate autonomously through different environments. By analyzing visual cues, the vision-based navigation method allows UAVs to adapt to unstructured environments, recognize and identify obstacles with precision, and avoid them, thereby ensuring safe operations [28].

Table 1 lists the numerous advantages of vision-based navigation, such as comprehensive visual data, as well as the challenges, such as performance degradation under adverse weather conditions and the need for adequate lighting for optimal functioning. The objective of the current study is also to automate an inspection using a collaborative UAV–UGV system. The increasing complexity and scale of modern industrial facilities have increased the need for efficient, accurate, and safe inspection solutions. Traditionally, manual inspections have been the primary method of assessing the condition of equipment. However, these manual processes are fraught with challenges, including the risk of human error and the dangers associated with operating in hazardous areas. For instance, manual gauge readings are often performed periodically, requiring human operators to record the values of meters of various shapes, scales, and display types. These meters are sometimes located in hazardous or hard-to-reach areas. The task of reading these gauges is further complicated by environmental factors, such as intense electromagnetic radiation and the risk of electric shock, which make manual inspections labor-intensive and potentially dangerous. To address these challenges, we explore the development of a UAV–UGV collaborative system that can autonomously perform the inspection round, leveraging computer vision for navigation and deep learning for meter readings. Research in this domain has focused on improving the accuracy and reliability of meter gauge reading under various conditions. For example, Wu et al. [29] introduced a high-precision automatic pointer meter gauge reading system designed to address challenges such as low-light conditions, which can degrade the accuracy of visual data processing. Similarly, Hong et al. [30] developed an image-based water meter gauge reading solution to overcome challenges in complex environments, including dynamic lighting, occlusions, and featureless surfaces, which often hinder the performance of vision-based systems. Similarly, the study in [31] proposed a text detection-based algorithm to enhance the robustness of pointer meter gauge readings, and Fang et al. [32] explored a meter gauge recognition algorithm tailored for inspection robots. These innovations underscore the increasing demand for multi-agent robotic systems in industrial applications, showcasing their potential to enhance safety, minimize human error, and improve the overall efficiency of inspection operations. Table 2 provides a summary of the key findings and methodologies for related work.

## 3. System Overview

**Figure 3:**
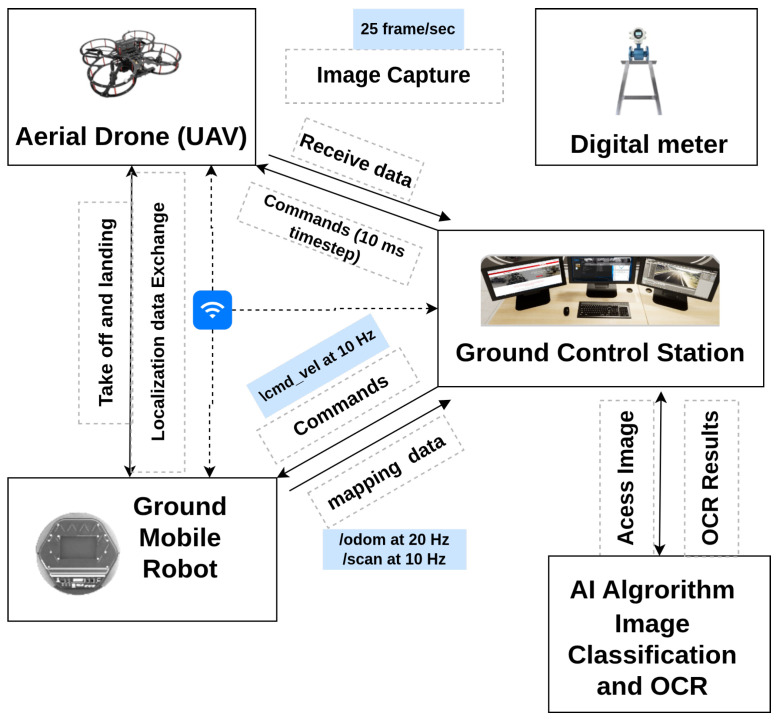
System architecture of the proposed multi-domain robotic inspection framework. The diagram illustrates the interaction between the UAV, the UGV, and the GCS, highlighting data exchange over TCP/IP and ROS topics for real-time inspection and decision-making.

This section presents an overview of the system used in this research, highlighting its key components, their integration, and the workflow designed to achieve the experimental objectives. The proposed system, as shown in Figure 3, integrates a TurtleBot 2 robot and a QDrone 2 to collect and process data from a transmitter sensor autonomously. The TurtleBot 2 serves as the carrier for the QDrone 2 and navigates autonomously towards the transmitter sensor. It features LiDAR technology to generate a map of the surrounding environment using the SLAM algorithm. The pre-map is then used to navigate in the operational environment and avoid obstacles. Throughout its mission, the robot maintains continuous communication with the main Ground Control Station (GCS), regularly reporting its status and condition. Upon reaching the gauge, the TurtleBot 2 robot sends an arrival notification to the GCS, which subsequently commands the QDrone 2 to take off.

The QDrone 2 operation involves taking off from the UGV, capturing a high-resolution image of the transmitter sensor to extract its reading, and landing securely on the robot. The captured image is transmitted to the GCS, where an AI algorithm processes it to extract the sensor’s reading, such as text or numerical data. Finally, the extracted data are stored in the server database, enabling future analysis and reporting. This system effectively combines ground and aerial robotic platforms with AI-driven data processing to achieve seamless and automated sensor data collection in dynamic industrial environments.

Accurate mapping and localization are essential for enabling fully autonomous operation within complex, GPS-denied industrial environments. To support this, the UGV utilizes a LiDAR-based SLAM system that generates real-time occupancy grid maps of the environment, facilitating dynamic navigation. The SLAM module is implemented using the ROS SLAM Toolbox and operates onboard the UGV, allowing it to localize its position relative to the environment, detect obstacles, and plan collision-free paths. This mapping functionality forms the foundation for path planning, obstacle avoidance, and provides the localization framework required for precision UAV landing and task coordination.

## 4. Experimental Setup

### 4.1. Selection of UAV System

**Figure 4:**
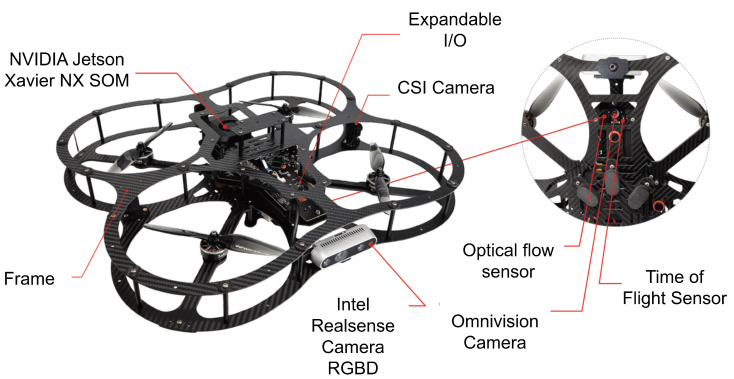
Sensor suite of the Quanser QDrone 2 used for vision-based landing, including the NVIDIA Jetson NX, CSI camera, optical flow sensor, and range sensor, integrated into the onboard flight controller for autonomous navigation and landing.

When considering suitable platforms for our research on advanced UAV control systems, two prominent options emerged: the DJI Mavic [33] and the Quanser QDrone 2 [34], whose specs are shown in Table 3. The DJI Mavic 2 Pro offers advantages such as low noise emission (approximately 75 dBA) [35], making it suitable for indoor applications like manufacturing plants. It is compliant with OSHA noise level regulations and boasts high-resolution imaging capabilities. Its primary focus is on consumer-grade photography and videography. This translates to limited access to low-level control and a closed-source architecture, hindering its suitability for in-depth research and development of flight control systems. In contrast, the Quanser QDrone 2, specifically designed for research and education, offers unparalleled access to low-level control and integrates seamlessly with development tools such as Quanser Quarc and MATLAB/Simulink. These features enable researchers to design, test, and implement custom control strategies, including vision-based tasks such as autonomous landing using AprilTags (See Figure 4). Furthermore, the QDrone 2 integrates well with localization systems like OptiTrack, providing accurate ground-truth data for experimental validation. Its flexible design accommodates custom payloads and configurations, making it a versatile platform for diverse research needs. Table 3 shows the comparison between the DJI Mavic 2 Pro and the Quanser QDrone 2.

### 4.2. Preliminary Modification to the Standard Robot

**Figure 5:**
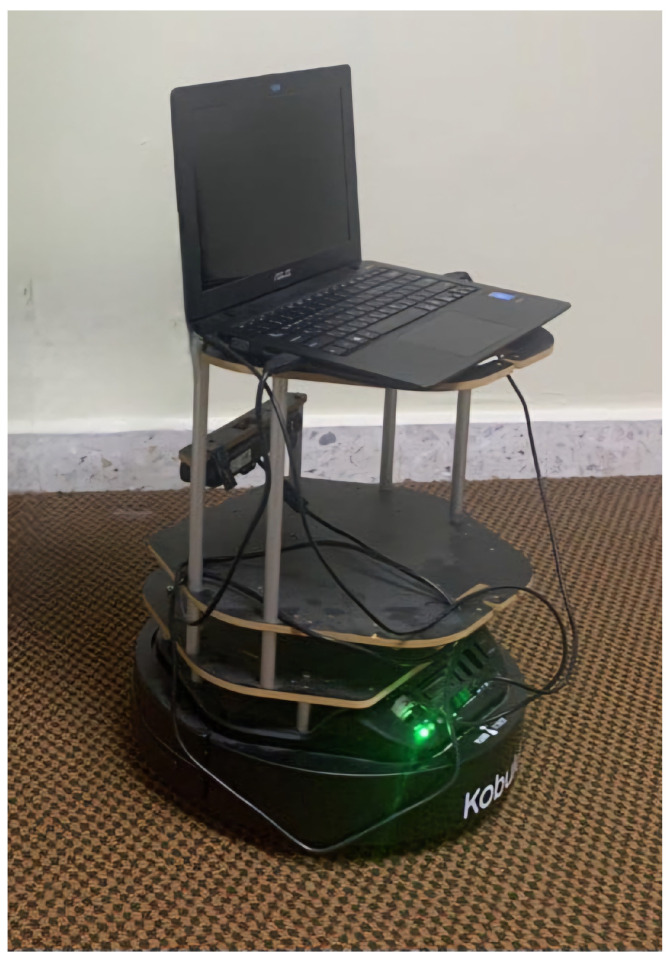
Standard configuration of the TurtleBot 2 platform before modification, showing the multi-layer structure and onboard PC used in initial experiments.

**Figure 6:**
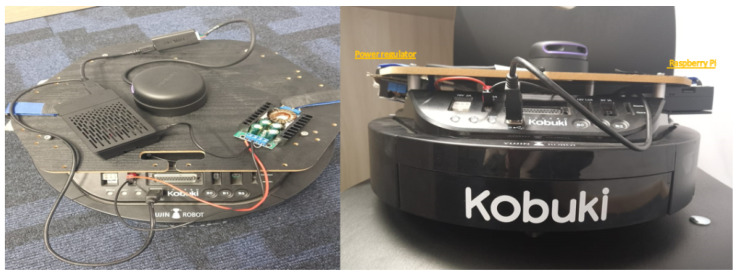
Modified TurtleBot 2 design with flattened top surface for drone landing. The onboard PC was replaced by a Raspberry Pi, and a LiDAR sensor was integrated to enhance mapping performance in indoor environments.

The original design of the TurtleBot 2 includes multiple top layers supported by beams, which create challenges for stable drone landing (See Figure 5). To address this, the top layers needed to be removed to provide a more stable and lower landing surface for the drone. However, one of these top layers houses the onboard PC, which is responsible for controlling the robot. Removing this layer necessitated replacing the onboard PC with a smaller, more compact controller, such as a Raspberry Pi, which is better suited to the modified TurtleBot design. The standard Intel RealSense D435 camera on the TurtleBot 2 has significant limitations under varying indoor lighting conditions, such as bright light or direct sunlight, which can cause blind spots during mapping. Additionally, the camera’s narrow field of view and limited capacity to detect small or low-lying obstacles further reduce the robot’s navigation accuracy. The Intel RealSense D435 camera provides detailed surface information; however, its resolution for distance measurements is insufficient for precise indoor mapping tasks. As an alternative, the integration of a LiDAR sensor was proposed (See Figure 6), as it provides higher accuracy in distance measurement and better addresses the limitations of the Intel RealSense D435 camera. The replacement of the onboard PC with a Raspberry Pi, along with the substitution of the Intel RealSense D435 camera with a LiDAR sensor, posed another challenge regarding power supply. Both the Raspberry Pi and the LiDAR sensor require 5 volts to operate, while the TurtleBot 2’s onboard power supply delivers 12 volts, initially intended for the onboard PC. To manage this discrepancy, a power converter was recommended to ensure the new components function properly without exceeding their voltage limit.

### 4.3. Lab Setup

To validate the proposed system under physical conditions, an indoor experimental environment was configured to simulate realistic industrial inspection scenarios. The laboratory setup integrates a UGV (TurtleBot 2), a QDrone 2 UAV, and a GCS, with physical fixtures such as meter gauge targets and environmental obstacles arranged to mimic constrained and cluttered plant environments.

**Figure 7:**
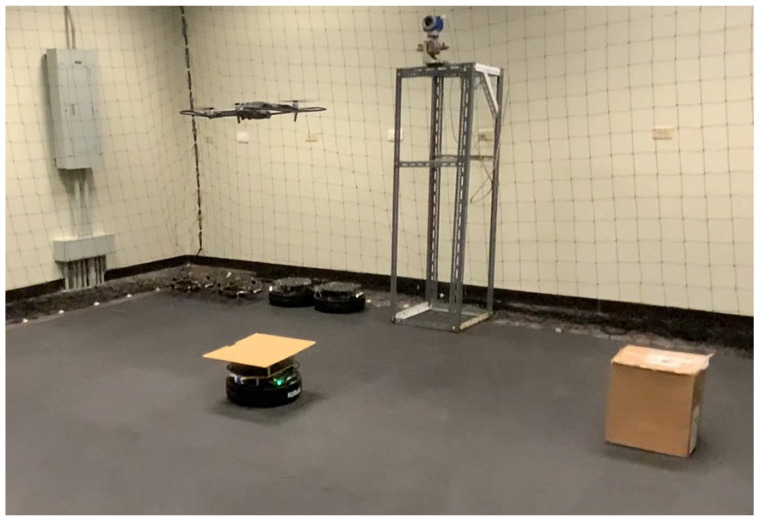
Experimental laboratory setup for testing UAV–UGV collaboration during automated meter gauge inspection. The environment includes fixed inspection stands and markers to simulate real-world industrial conditions.

Figure 7 shows the physical layout of the lab. The experimental arena was configured with both structured and unstructured elements, including cardboard boxes and vertical structures placed to obstruct the line of sight and movement. This configuration was designed to test the SLAM-based mapping and autonomous navigation capabilities of the UGV, as well as to challenge the vision-based landing and positioning accuracy of the UAV. A fixed-height stand with a mounted digital meter gauge was included as the inspection target, allowing the UAV to capture images at a consistent altitude. The GCS (shown in Figure 3) managed communication and coordination between the agents, processed sensor data, and served as the interface for real-time monitoring and control.

Software-wise, the lab experiments utilized the same ROS–Simulink integration as the simulation environment. SLAM was tested using the SLAM Toolbox and visualized in RViz, while the UAV’s flight control and landing routines were executed using Simulink models deployed via Quanser Quarc. Ground-truth data for UAV and UGV trajectories were obtained using the OptiTrack motion capture system.

This physical setup allowed for end-to-end validation of:

- SLAM performance in obstacle-rich environments,
- Autonomous navigation and obstacle avoidance by the UGV,
- Precise UAV landing using vision-based detection,
- System coordination under event-triggered socket communication,
- AI-based meter reading using real imagery captured in the lab.

Overall, the lab environment provided a controlled yet realistic testbed that bridges the gap between simulation and field deployment.

## 5. Development and System Implementation

Building on the simulation and lab configurations described in the previous sections, this section details the implementation of the system’s key functional components, including mapping and navigation on the UGV, as well as vision-based landing and control on the UAV.

### 5.1. UGV Mapping and Navigation

SLAM plays a pivotal role in enabling autonomous navigation within complex indoor environments. In this work, the TurtleBot 2 UGV was equipped with a 2D LiDAR sensor and configured to perform SLAM using the SLAM Toolbox within the ROS environment. This allowed the robot to construct occupancy grid maps of its surroundings and localize itself in real time during inspection missions.

**Figure 8:**
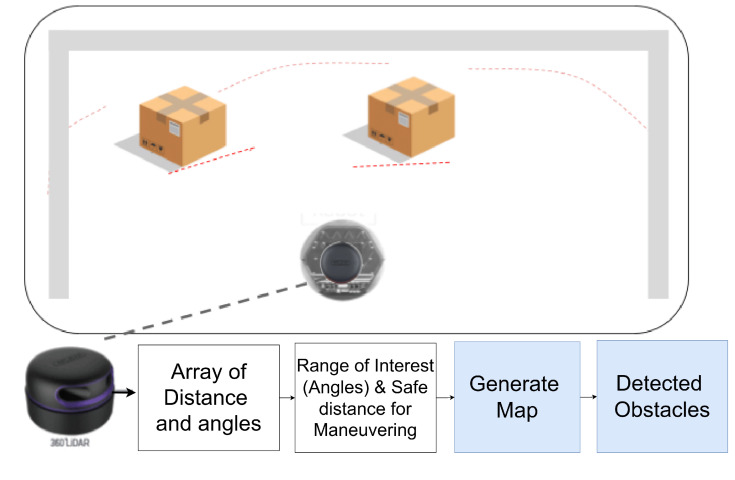
Illustration of LiDAR-based obstacle detection and mapping using a TurtleBot 2 UGV. The figure shows the data processing pipeline from laser scan measurements to map generation, with identified obstacles and safe navigation zones highlighted.

Figure 8 illustrates the LiDAR-based mapping and obstacle detection setup. The sensor captures 360-degree scan data, which is processed to extract distance and angle information. These data are then used to generate the map, define navigation constraints, and plan safe paths to target locations. The mapping system ensures reliable obstacle avoidance and precise maneuvering, which is essential for positioning the UAV for inspection tasks.

The SLAM Toolbox [36], integrated with ROS, was selected as the primary algorithm for both simulation and real-world tests. Key parameters were carefully tuned to enhance performance:

- **Map resolution**: 0.05 m per grid cell, balancing accuracy and computational load.
- **Update rate**: Adjusted for real-time responsiveness during continuous scan processing.
- **Sensor input**: Real-time laser scan data from the onboard RPLiDAR sensor.

These configurations allowed consistent and accurate map generation across diverse test scenarios. Visualization was conducted using RViz, which provided real-time feedback on mapping progress, localization accuracy, and obstacle detection. RViz also proved helpful in tuning parameters by highlighting misalignments and inconsistencies during test runs. Prior research has demonstrated RViz’s effectiveness in SLAM development and validation [37,38].

SLAM performance was validated using the Gazebo simulation environment and RViz visualization tools. The SLAM implementation was tested in Gazebo, a virtual environment that simulates complex real-world situations [39]. A cluttered environment was constructed with nine cylindrical obstacles. The TurtleBot 2 began from a fixed pose and was tasked with autonomous exploration. The SLAM Toolbox, combined with the LiDAR feed, enabled the robot to incrementally build a consistent map and localize itself while avoiding collisions. The performance was optimized by adjusting parameters, including map resolution and scan matching tolerances. RViz, a 3D visualization tool within ROS, provided real-time feedback during the simulation, enabling us to dynamically monitor the robot’s movement, sensor readings, and map generation. The TurtleBot 2 successfully navigated a cluttered test arena, demonstrating accurate localization and map consistency. RViz visualization confirmed successful mapping and obstacle navigation throughout the simulation [40].

While the present work focuses on a single-UGV implementation within a controlled lab environment, future extensions could involve multi-robot or bandwidth-constrained deployments. For example, Zhang et al. [21] proposed a multi-robot SLAM framework that uses compressed occupancy grid maps, reducing communication bandwidth by up to 99% while maintaining localization accuracy. Although our system does not require distributed SLAM or inter-robot map sharing, such bandwidth-aware strategies represent promising avenues for scalable swarm-based industrial applications.

### 5.2. UAV Vision-Based Landing and Localization

For the UAV component, precise landing is essential to ensure the safe return of the QDrone 2 after inspection missions. This was achieved using a vision-based approach that leverages AprilTags for visual fiducial tracking. The UAV’s onboard camera detects and tracks AprilTags mounted on the top surface of the UGV, enabling accurate pose estimation relative to the landing platform. Landing control was implemented using a PID and PIV architecture, configured in Simulink and deployed via the Quanser Quarc platform. The UAV uses visual data to align itself with the AprilTag, adjusts its horizontal position and yaw, and initiates controlled descent. To ensure operational safety, a watchdog system monitors critical parameters, including communication integrity, battery voltage, and sensor health, and triggers emergency routines in the event of anomalies.

To support ground-truth validation, the localization system was augmented with an OptiTrack motion capture system, which provides ground-truth position data for experimental validation. This setup allows precise tracking of both UAV and UGV movements during the landing process. The control system, including image acquisition, AprilTag detection, pose computation, and motor command generation. Real-time synchronization between the Simulink model and the ROS-based UGV system was achieved via TCP/IP socket communication. An event-triggered signaling mechanism was used to minimize bandwidth overhead and ensure reliable coordination between agents (UAV and UGV).

Although this vision-based landing approach performed reliably in controlled indoor conditions, its effectiveness may degrade under varying lighting, camera tilt, or partial marker occlusion. Future work will explore adaptive vision models, markerless detection, and multimodal sensor fusion (e.g., fusing visual tags with inertial or range sensor data) to improve robustness in complex industrial settings.

**Figure 9:**
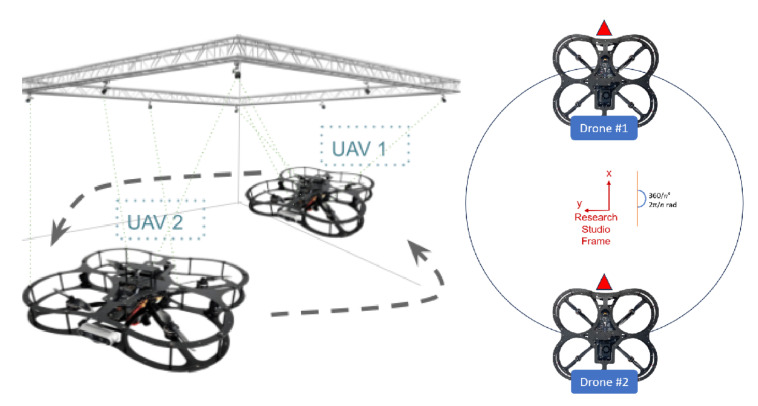
Evaluation of QDrone 2 flight tracking accuracy during circular trajectory tests using the OptiTrack motion capture system. The figure demonstrates real-time position tracking and control validation, which are critical to the feasibility of swarm operations.

**Figure 10:**
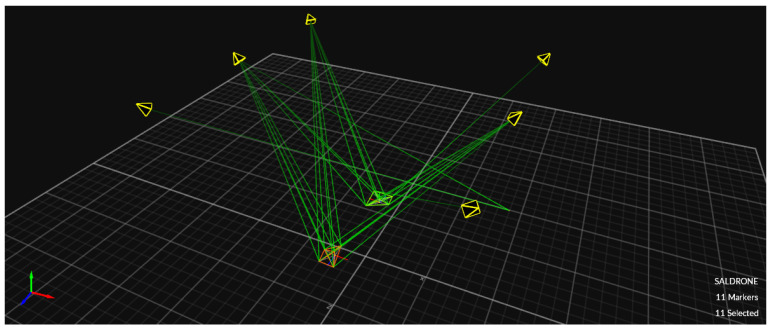
OptiTrack motion capture system setup comprising six infrared cameras used to track the position and orientation of two QDrone 2 units. Teal markers indicate real-time tracking vectors used for ground-truth validation.

**Figure 11:**
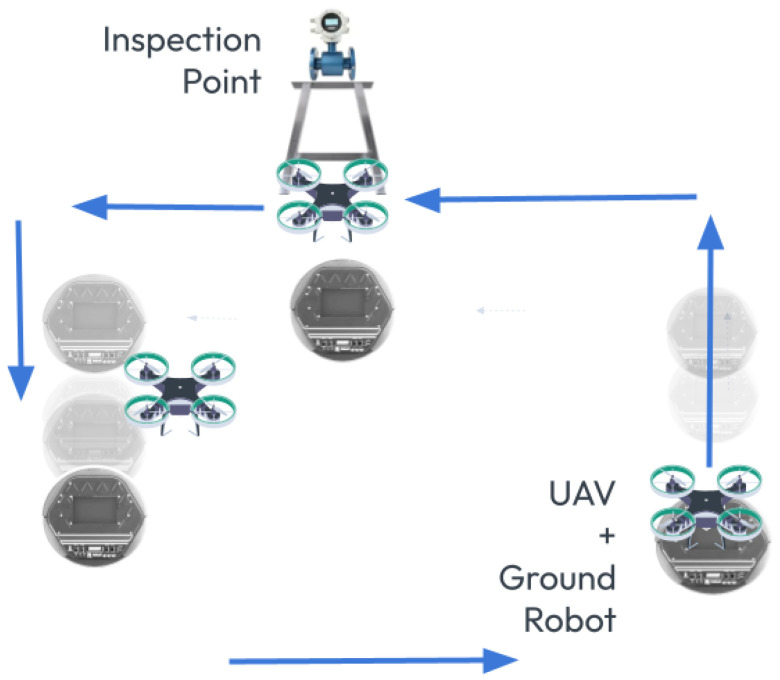
Conceptual scenario showing coordinated task execution between a UAV and a UGV. The UAV performs an overhead inspection while the UGV transports and positions the platform. Arrows indicate motion paths and task sequence for both robots during collaborative operation.

To isolate software issues from hardware malfunctions, this work utilized two QDrone models (1st and 2nd) to validate the core functionality of the Swarm-QDrones operation process. By observing the QDrone’s behavior during flight, specifically its ability to maintain a circular trajectory within the workspace as illustrated in Figure 9 and Figure 10, the real-time position tracking performance of each QDrone is evaluated in a horizontal reference frame. The measured position tracking response of each QDrone is compared against the desired position tracking response to assess the system’s accuracy. These findings are highly relevant to our work, as they demonstrate the fundamental requirements for building reliable and effective swarm systems. A conceptual coordinated task sequence is illustrated in Figure 11.

### 5.3. Communication Between Mobile Robot and Drone System

In multi-domain robotic systems composed of heterogeneous agents such as UAVs and UGVs, achieving reliable and efficient communication is critical for cooperative task execution. This section describes the implementation of a bidirectional, event-triggered communication framework that enables coordination between the UGV, the UAV, and the GCS.

The UAV and UGV systems were developed using different platforms: the UGV runs ROS on a Linux-based system, while the UAV control algorithms are implemented using MATLAB Simulink on a Windows-based system. This difference in operating environments necessitated a cross-platform communication solution capable of low-latency operation, time synchronization, and data integrity.

To address these requirements, a TCP/IP socket-based communication protocol was implemented [41]. The UGV acts as a client, while the GCS hosts the server application that also interfaces with the UAV. Data exchange is event-triggered rather than continuous to reduce bandwidth usage and energy consumption, particularly on the UAV side.

A typical communication sequence begins when the UGV reaches a designated inspection point and publishes its position data to the ROS topic /odom. These data are captured by a custom Python ROS node, formatted into a structured JSON object containing the x, y, and yaw values, and sent to the GCS via TCP socket. The GCS processes the received data and relays the required instructions to the UAV. Once triggered, the UAV executes a predefined mission sequence including takeoff, navigation, image capture, and landing.

To ensure consistent timing between systems, the Simulink model running on the UAV subscribes to the ROS /clock topic. This alignment ensures accurate execution of time-dependent logic blocks in Simulink and proper synchronization with the rest of the system.

**Figure 12:**
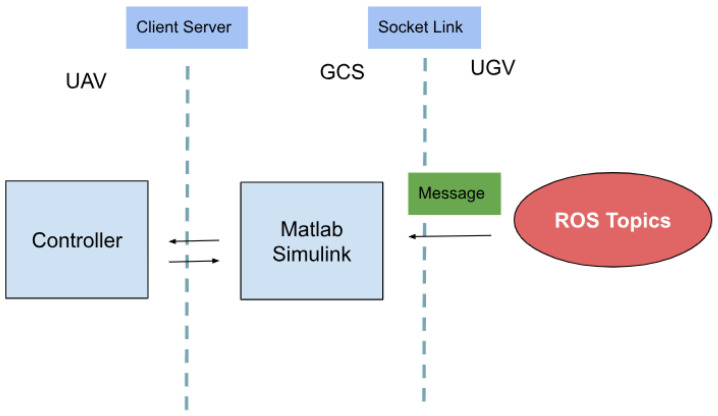
Communication architecture for integrating UAV control in Simulink with ROS-based UGV coordination. The setup includes socket interfaces and ROS topics enabling synchronized event-driven collaboration between UAV, UGV, and the Ground Control Station.

The communication architecture is shown in Figure 12, which highlights the interaction between the ROS environment, Simulink model, and the socket interface.

The communication operates on a static IP setup within a local area network, with a typical data rate of 10–20 Hz. Data integrity is ensured by acknowledgment mechanisms implemented on the server side. Compared to conventional leader-follower approaches that rely on continuous polling or centralized control, the proposed architecture is modular, scalable, and event-driven. This minimizes unnecessary communication overhead and power usage, while maintaining real-time performance. The system enables the efficient integration of heterogeneous agents using lightweight protocols across various platforms.

The communication logic for sending data from the UGV side to the UAV side is summarized in Algorithm 1.
**Algorithm 1** Event-Based Communication between UGV and UAV via GCS1:**Input:** UGV position data from /odom ROS topic2:**Output:** UAV mission triggered upon UGV arrival3:**UGV Node (ROS/Linux):**4:Start navigation toward inspection waypoint5:**while** UGV is navigating **do**6:   **if** Waypoint is reached **then**7:     Extract position (x,y,yaw) from /odom8:     Format position as JSON object9:     Send data via TCP socket to GCS (Windows)10:   **end if**11:**end while**12:**GCS Server (Windows):**13:Initialize socket server (static IP and port)14:**while** Receiving data from UGV **do**15:   Parse and validate received JSON16:   Log message and trigger UAV mission in Simulink17:   Send acknowledgment to UGV18:**end while**19:**UAV Controller (Simulink/Windows):**20:Subscribe to ROS /clock for synchronization21:Wait for mission trigger signal from GCS22:Execute mission: *takeoff* → *hover* → *capture image* → *land*23:**Shutdown:**24:Close all socket connections and stop mission routines safely

### 5.4. Vision-Based Landing

#### 5.4.1. Vision-Based Localization and Tag Detection

According to [42], the primary challenges in autonomous UAV landing include precise estimation of both the UAV’s and the landing platform’s positions, as well as ensuring robust trajectory tracking in the presence of disturbances and uncertainties. To address these challenges, several advanced control strategies have been proposed. One approach involves using a PD controller for attitude regulation, combined with vision-based tracking, which enables autonomous landing on a stationary platform. Another method integrates feedback linearization, utilizing an inner loop for attitude control and an outer loop for velocity and altitude control, along with 2D tracking, to facilitate landing on a moving platform. Additionally, extended backstepping nonlinear controllers, often used in conjunction with a tether, have been shown to enhance stability during landing. 2D vision markers have been utilized to guide a quadcopter in a specific direction in [43]. The quadcopter’s camera reads QR codes, enabling it to follow a desired trajectory. However, the camera’s ability to extract information is influenced by factors such as the distance from the object, the drone’s altitude during aerial operations, and the camera’s focal length [44].

Many studies, such as [42], emphasize the critical role of computer vision in autonomous UAV landing techniques. Various image-based methods have been developed to track landing platforms with greater precision. One approach uses image-based visual servoing (IBVS) to track a platform in two-dimensional image space. At the same time, another integrates dynamic IBVS with translational optimal flow to enhance velocity measurement accuracy. Additionally, visual fiducial systems, such as AprilTags, have been combined with IMUs and GPS on moving targets traveling at high speeds, enabling robust tracking in dynamic environments. These advances in computer vision are crucial for enhancing the reliability and performance of autonomous UAV landing systems, particularly in complex and high-speed scenarios.

#### 5.4.2. Motion Capture Integration and Control Architecture

**Figure 13:**
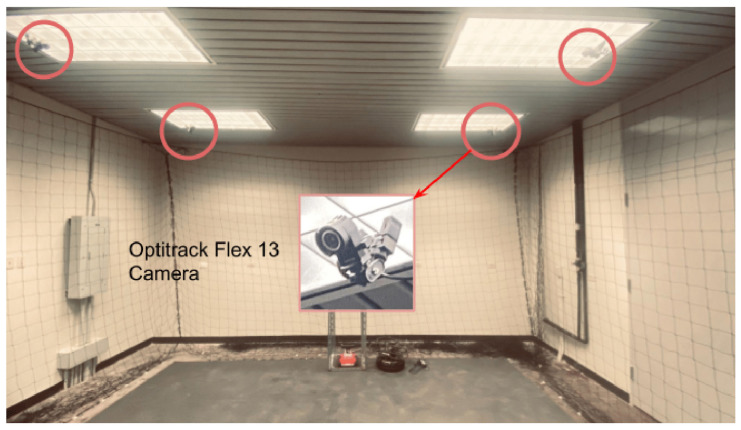
OptiTrack motion capture system with six infrared cameras used to measure the UAV’s ground-truth position and orientation. This system provides real-time, high-precision localization data for validating autonomous navigation and landing performance.

**Figure 14:**
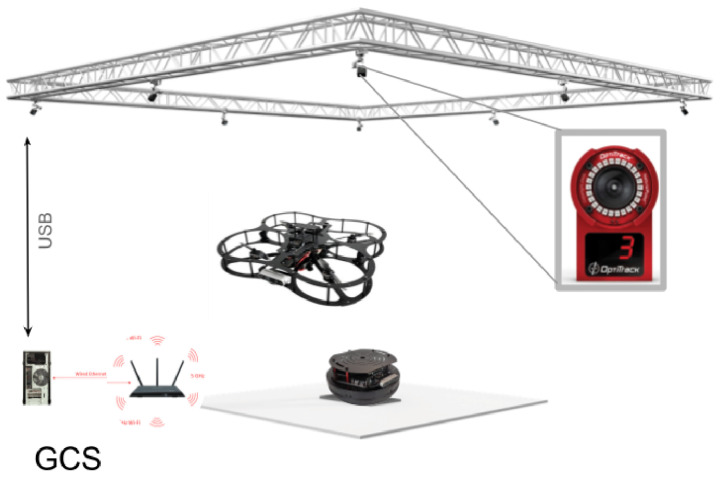
Comparison of UAV altitude measurements from onboard range sensors and the OptiTrack ground-truth system. The OptiTrack data serves as a reference to evaluate the accuracy and noise levels of the onboard sensor readings during flight.

The physical lab features an OptiTrack motion capture system, which provides real-time, high-precision localization data crucial for inspection tasks. By using multiple infrared cameras, the system tracks reflective markers on the QDrone, delivering accurate positional and orientation data. This localization serves as a reliable ground truth, ensuring that the drone can precisely navigate and align itself for inspection points. For inspection tasks, the OptiTrack system illustrated in Figure 13 ensures the QDrone maintains stable flight and accurate positioning while interacting with dynamic environments. A comparison of altitude measurements from the onboard range sensor and the OptiTrack ground-truth system is shown in Figure 14. The integration of OptiTrack with tools like Simulink facilitates real-time data exchange and control, allowing the QDrone to effectively fulfill inspection requirements such as monitoring digital meters or capturing data from specific targets.

The PID controller is widely recognized for its simplicity and robustness in handling a wide range of operating conditions. The proportional term directly responds to the current error, the integral term helps reduce steady-state errors by accounting for past errors, and the derivative term predicts future errors, improving system stability and reducing overshoot. Despite its effectiveness in linear systems, the PID controller struggles when applied to systems with nonlinearities, uncertainties, or those lacking precise mathematical models, resulting in degraded performance [45].

#### 5.4.3. System Implementation, Safety, and Autonomous Landing Logic

**Figure 15:**
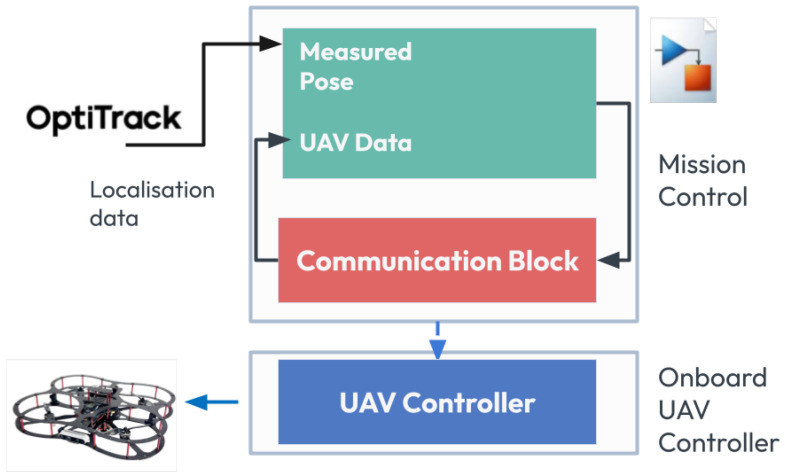
Overview of the Simulink control model used for vision-based UAV landing. Key components include position and attitude control loops, AprilTag detection logic, and command signal generation for autonomous descent.

For the Quanser QDrone 2, PID control can be utilized for fundamental flight dynamics, such as stabilization and position control. However, the QDrone 2’s control architecture also includes PIV controllers, as shown in Figure 15, which offer specific advantages over the traditional PID approach. The manufacturer provides pre-configured PIV controller parameters, where the derivative term operates directly on the calculated velocity, rather than the error signal, unlike in PID control. This distinction makes the PIV controller particularly effective in managing systems with un-modeled friction effects. By adding an integrator term, the PIV controller helps minimize steady-state errors, enhancing the system’s performance under various flight conditions.

The PIV controller is conceptually similar to the PID controller but is specifically designed to enhance performance in dynamic environments, such as those encountered by UAVs. In the case of the QDrone 2, which is primarily used for research and control experiments, the PIV controller’s ability to handle velocity-based control makes it a suitable choice for more advanced applications. The position data, captured by OptiTrack’s motion capture system can be fed into the control algorithm, enabling more precise feedback for position and velocity control. By utilizing these data, the UAV can adjust its flight path in real time based on its actual position relative to the target.

**Figure 16:**
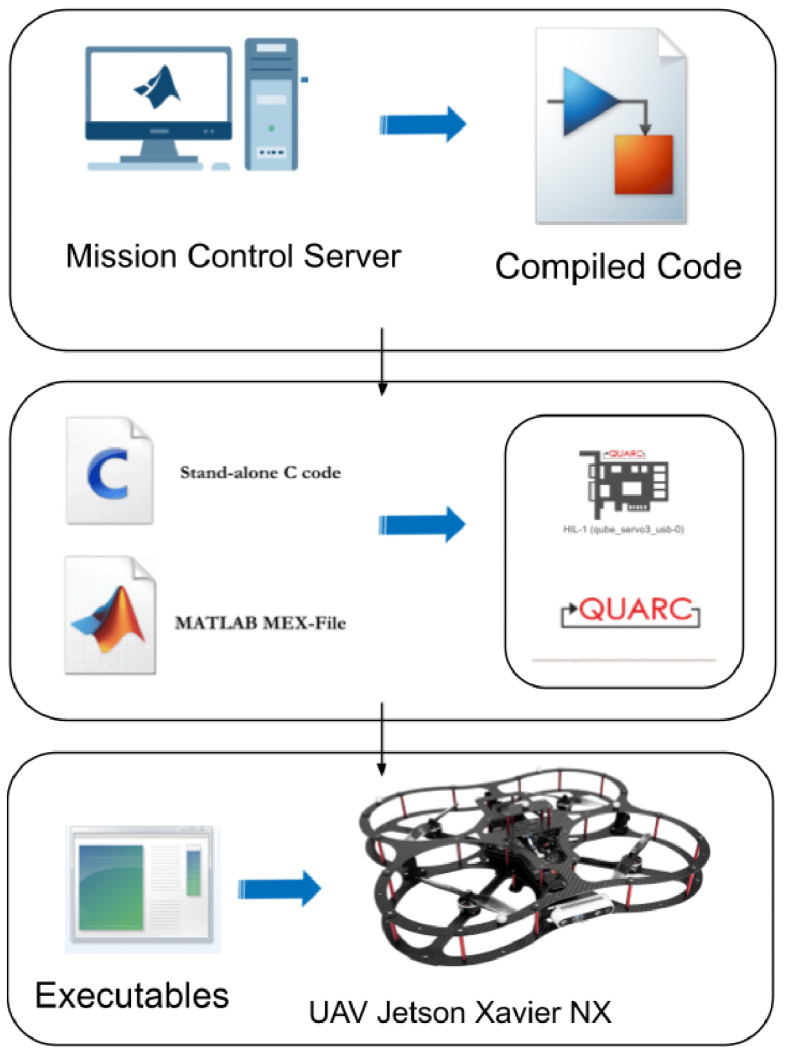
Simulink–Quanser Quarc integration used for developing and deploying control algorithms to the QDrone 2. The model enables real-time execution by generating embedded C/C++ code directly from the Simulink environment.

Figure 16 shows the Quanser Quarc platform to facilitate the development and deployment of a control algorithm for the QDrone 2. The algorithm is designed and simulated within the Simulink environment [46]. Subsequently, Quanser Quarc translates the Simulink model into C/C++ code optimized for the QDrone 2. This generated code is then directly deployed to the QDrone 2 for real-time execution. This approach streamlines the development process by seamlessly integrating simulation, code generation, and real-time implementation.

**Figure 17:**
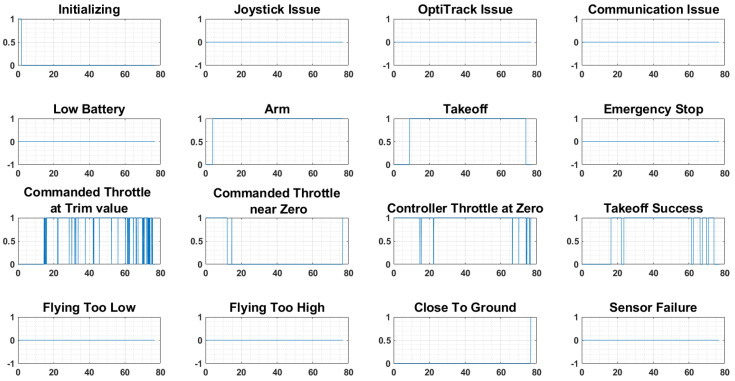
Built-in watchdog monitoring system on the QDrone 2 platform, showing the activation of 14 diagnostic triggers for detecting software or hardware anomalies. These watchdogs enhance UAV safety by enabling preemptive fault handling.

As UAVs grow more complex, ensuring their safety becomes paramount. The potential for cases like a Loss of Control (LOC) event necessitates robust safety mechanisms. This has spurred research into fault-tolerant control strategies and safety features, although it is not as advanced as what was developed in [47], the watchdogs implemented in Quanser QDrone systems, which monitor critical flight parameters indicated in Figure 17 and trigger safety protocols to prevent accidents. The Quanser QDrone 2 incorporates a comprehensive watchdog system to ensure safety and reliability during research and inspection tasks. It monitors critical parameters, including communication with the ground station, battery health, sensor performance, flight stability, and environmental factors. When irregularities are detected, the system can take corrective actions, such as engaging alternative sensors, initiating hover mode, or executing a controlled landing. This functionality supports safe and uninterrupted operation, particularly in complex scenarios, making it well-suited for experimental and inspection activities in diverse environments [47].

**Figure 18:**
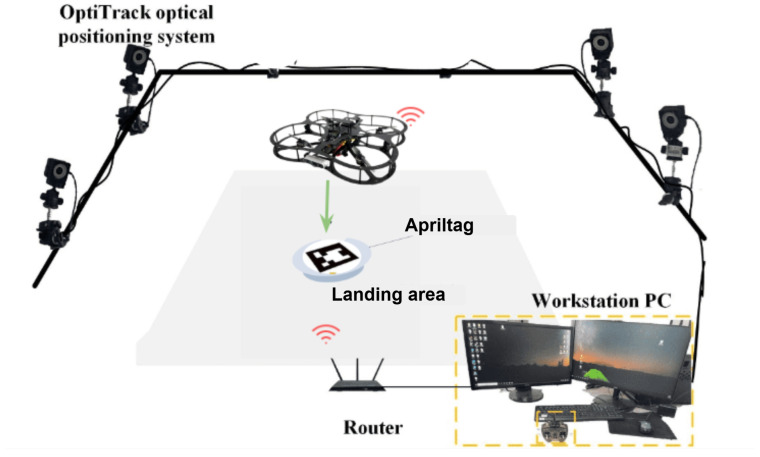
Experimental setup for vision-based UAV landing. The system integrates a QDrone 2, OptiTrack motion capture cameras, and AprilTag-equipped landing platforms to support real-time visual guidance and precision descent control [13].

**Figure 19:**
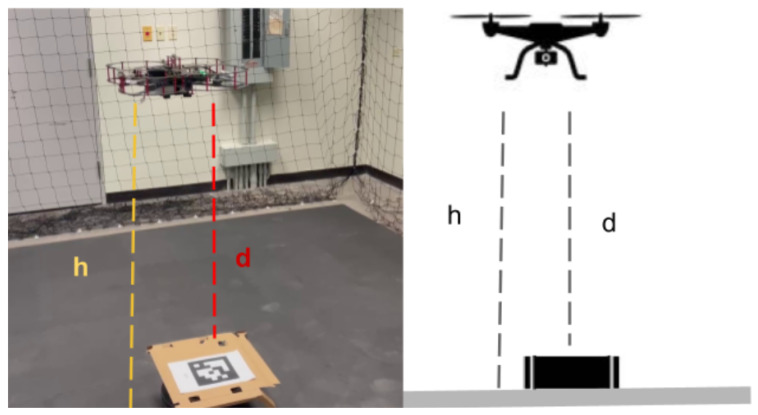
Vision-based UAV landing process showing AprilTag detection and descent, with annotated comparison of altitude estimates: OptiTrack ground-truth height (h) versus onboard range sensor distance (d).

Figure 18 depicts the experimental platform for a UAV–UGV system incorporating vision-based landing capabilities. The system utilizes a QDrone quadrotor, an OptiTrack motion capture system, and a ground workstation PC. The OptiTrack system, equipped with ten cameras, captures and processes the 3D position and orientation data of both the UAV and the UGV. Additionally, the UAV is equipped with a camera for visual navigation, enabling it to detect and localize AprilTags placed on the landing pad as described in Figure 18 and Figure 19. The ground workstation receives data from both OptiTrack and the UAV’s camera, processes this information, and generates appropriate control commands for the UAV, including those necessary for vision-based landing. Communication between the UAV and the ground station is facilitated through a 5 GHz WiFi network. This integrated system allows for precise localization, navigation, and autonomous landing of the UAV.

**Figure 20:**
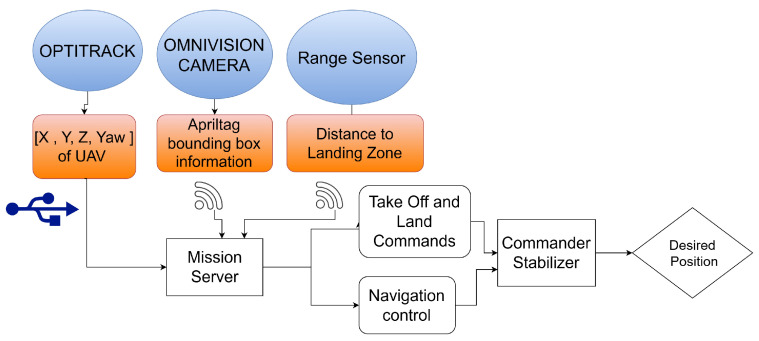
Data flow diagram of the vision-based UAV landing system, illustrating communication between onboard sensors, vision algorithms, control modules, and the ground station for mission execution.

This diagram in Figure 20 illustrates a UAV vision-based landing system. Key components include an OptiTrack system for precise position and orientation tracking, an Omni-vision camera for visual data acquisition and AprilTag detection, and a range sensor for distance measurement. The data from these sensors is processed by a Mission Server, which generates appropriate commands for the UAV, including takeoff, landing, and navigation control signals. A Commander module then translates these commands into control signals for the UAV’s actuators, while a stabilizer ensures stable flight. This integrated system enables accurate localization and, ultimately, a safe and autonomous landing of the UAV.

The vision based UAV landing routine is explained as in Algorithm 2.
**Algorithm 2** Vision-Based UAV Landing1:**Input:** Camera Feed, AprilTag Parameters, OptiTrack Data (Optional)2:**Output:** Smooth and Accurate Landing3:Initialize UAV sensors (camera, IMU, altimeter)4:Load camera calibration parameters5:Set PID gains for X, Y, Z, and yaw control6:Define landing thresholds (position error, descent rate)7:**while** AprilTag NOT detected **do**8:   Capture camera frame9:   Detect AprilTag using the tag detection algorithm10:   **if** AprilTag detected **then**11:     Extract tag center coordinates (u,v)12:     Compute pose (X,Y,Z,yaw) relative to UAV13:   **end if**14:**end while**15:**repeat**16:   Compute error between camera center and AprilTag center17:   Update UAV X, Y position using PID controller18:   Correct yaw error using PID controller19:**until** Alignment error < Tolerance20:**while** UAV altitude > Landing Threshold **do**21:   Measure updated pose of AprilTag (X,Y,Z,yaw)22:   Adjust UAV position (X,Y) to minimize error23:   Reduce altitude incrementally24:   **if** AprilTag lost **then**25:     Pause descent26:     Search locally for tag reacquisition27:   **end if**28:**end while**29:Align UAV precisely above AprilTag (fine-tune X, Y, yaw)30:Reduce descent speed for smooth landing31:Disarm motors once touchdown confirmed32:**if** OptiTrack available **then**33:   Compare landing position with OptiTrack ground truth34:   Log position error and alignment data35:**end if**

### 5.5. AI-Driven Meter Gauge Reading

Accurate identification of points of interest is crucial for autonomous systems, such as robots and drones, operating in complex environments. As robots and drones move through an environment, they need to identify points of interest autonomously. In this work, we use the digital numbers displayed on a meter gauge as a point of interest, as shown in Figure 30. This section explores the challenges encountered when developing an AI model that can accurately read and detect these values from images captured by the UAV. Detecting meter gauge values begins with data acquisition, where the UAV’s embedded camera captures images. To enhance the accuracy of the subsequent object detection stage, the captured data undergoes pre-processing to improve image quality.

The choice of AI models used on the QDrone 2 is critical. Typically, more compact models, like MobileNet or Tiny YOLO, are chosen because they are designed to be faster and use fewer resources. These models are helpful because they strike a balance between speed and accuracy, which is crucial for real-time tasks [48].

To extract digital meter readings, we run the AI model directly on the UAV, minimizing the risk of communication loss during the mission [49]. This reduces the need for continuous data transmission, saving bandwidth and enabling the drone to handle tasks such as real-time image recognition more efficiently. However, even with this approach, there is still a need to balance computational power with energy consumption, as UAVs have limited battery life. Maintaining this balance without sacrificing accuracy is a constant challenge, especially for real-time tasks where swift decisions are crucial.

The UGV played a supportive role in this setup. It features various sensors, including LiDAR, IMUs, and cameras, which enable it to map its environment, navigate around obstacles, and serve as a mobile guide for the UAV. This collaboration helps the UGV navigate complex areas and position itself accurately, such as when it needs to read a digital meter gauge. However, the coordination between the UGV and UAV relies on real-time communication, and any delays can result in errors. For example, if there is a lag in data transmission, it could lead to incorrect positioning of the drone or issues with image capture, thereby affecting the accuracy of the collected data. It is also important to consider the energy requirements of these communication protocols, as maintaining a constant connection can quickly drain the UAV’s battery. This collaboration helps UAVs reduce the required flight time to reach the point of interest.

One of the methods commonly used for accurate digit extraction is Optical Character Recognition (OCR). OCR is a system that converts the input text into machine-encoded format [50]. Researchers have developed and optimized several tools for OCR. More recently, researchers have applied Recurrent Neural Networks (RNN), Convolutional Neural Networks (CNN), and Long Short-Term Memory (LSTM) for improving OCR performance [51]. According to analysis performed by Beerten [52] on different techniques of OCR, Tesseract OCR and Paddle OCR performed better in digit extraction than other OCR techniques. Moreover, Paddle OCR supports more than 80+ languages [53]. In this research, the YOLO (You Only Look Once) object detection algorithm is employed to identify and localize digital meters within the processed images [54]. YOLO’s real-time performance and high accuracy make it a suitable choice for this application, enabling the UAV to detect and track meter gauges in dynamic environments efficiently. Additionally, the Paddle OCR algorithm [53] was utilized, which is one of the high-performing state-of-the-art OCR algorithms [52].

## 6. Results and Discussion

In this section, the results obtained from the implementation and testing of the UAV–UGV system for inspection tasks were analyzed and presented. The discussion provides insights into system performance, focusing on vision-based landing accuracy, computational efficiency during UAV operation, the collaborative dynamics between the UAV and UGV, and the integration of advanced perception techniques. Key metrics, including mapping performance, visualization through RViz, real-time localization, adaptability, flight stability, control accuracy, and trajectory tracking, were analyzed to validate the system’s effectiveness. Of particular importance are the results related to digital meter gauge reading, where YOLO-based object detection and OCR technologies were employed to detect and extract readings from meters during UAV operations accurately.

### 6.1. UGV Mapping and Navigation Performance

**Figure 21:**
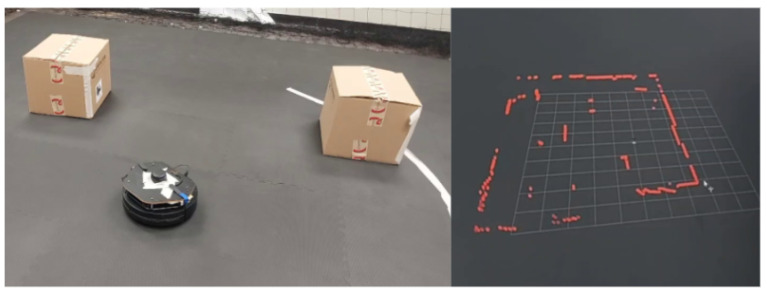
UGV (TurtleBot 2) equipped with RPLiDAR sensor (**left**) and corresponding scan visualization in RViz on the GCS (**right**), demonstrating real-time LiDAR-based mapping capability.

The mapping and navigation performance of the TurtleBot 2 was evaluated in both simulation and physical environments. The objective was to validate the SLAM-based autonomous navigation system implemented using the SLAM Toolbox in ROS, and to assess its reliability in cluttered industrial-like conditions. Figure 21 illustrates the UGV (TurtleBot 2) with the RPLiDAR and the corresponding RViz scan visualization.

**Figure 22:**
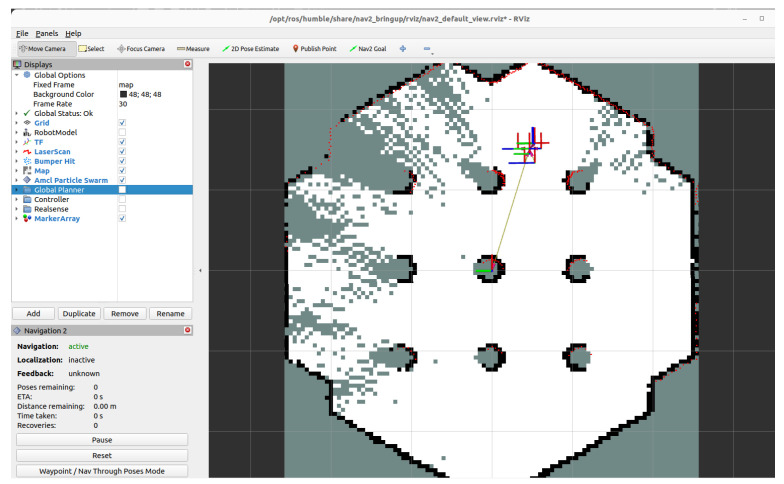
Incremental map construction using SLAM Toolbox in RViz during Gazebo simulation. The figure illustrates the robot’s active scan accumulation and environmental model generation in a virtual testbed.

In the simulation phase, a Gazebo environment was configured to replicate a constrained inspection arena. Nine cylindrical obstacles were placed to simulate common obstructions in industrial spaces. The TurtleBot 2 was initialized at a known pose and tasked with autonomously exploring the environment using LiDAR-based SLAM. Figure 22 shows the robot’s real-time mapping process visualized in RViz, and the resulting occupancy grid map generated during exploration. The robot demonstrated stable localization and consistent map generation throughout the simulation. Minimal pose drift was observed, and obstacle edges were clearly resolved in the generated map. The use of RViz enabled real-time monitoring of the robot’s pose, LiDAR scans, and map growth, providing visual confirmation of the SLAM algorithm’s effectiveness.

**Figure 23:**
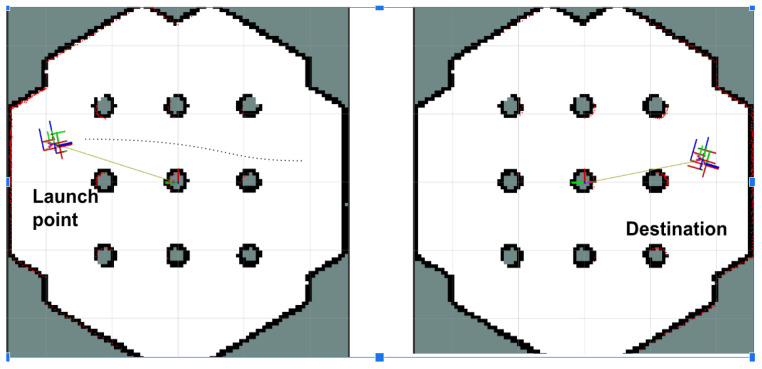
Snapshots from a Gazebo simulation where a TurtleBot 2 navigates a hexagonal arena populated with cylindrical obstacles. The scenario evaluates obstacle avoidance and path planning using LiDAR and SLAM in a cluttered environment.

**Figure 24:**
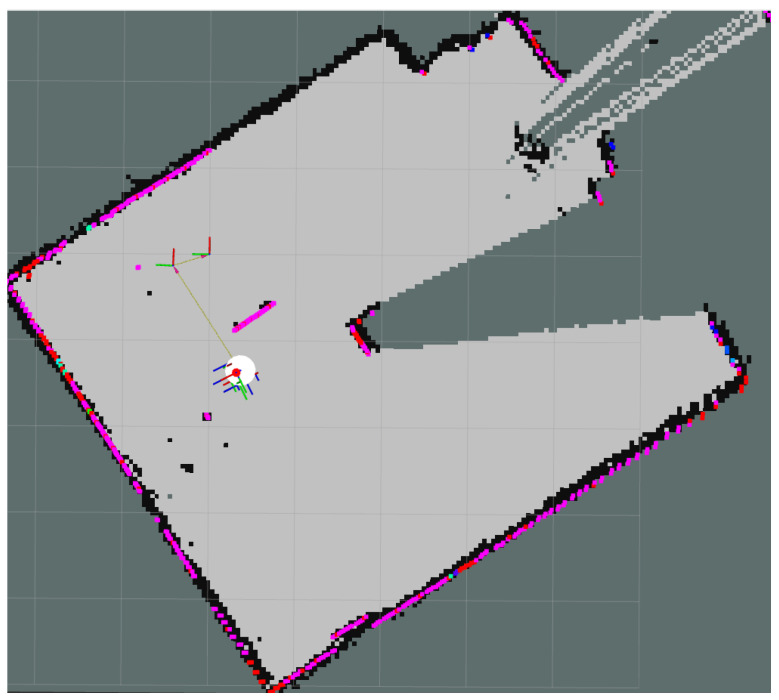
Real-time SLAM visualization using RViz during laboratory testing with the modified TurtleBot 2. The figure shows sensor feedback, robot pose estimation, and the dynamically generated map of the environment.

Figure 23 illustrates Gazebo snapshots of a TurtleBot 2 navigating a hexagonal arena populated with cylindrical obstacles to evaluate LiDAR- and SLAM-based obstacle avoidance and path planning. Figure 24 presents the results from the physical lab experiments, using the controlled setup described in Section 4.3. The robot successfully navigated the obstacle-filled environment and built a complete and accurate map of the space using real-world sensor input. The SLAM Toolbox parameters, including scan matching tolerances and update frequency, were reused from simulation and proved robust without requiring re-tuning. SLAM performance was evaluated qualitatively based on map completeness, feature alignment, and robot pose consistency. The system consistently identified obstacles and free space with sufficient resolution (0.05 m/cell) for navigation purposes. Additionally, no primary loop closure or alignment failures were observed, demonstrating the reliability of the SLAM pipeline for real-time localization of UGVs in indoor environments. These results confirm that the implemented SLAM system can support autonomous navigation in cluttered inspection settings and provides a reliable foundation for coordination with the UAV platform.

**Figure 25:**
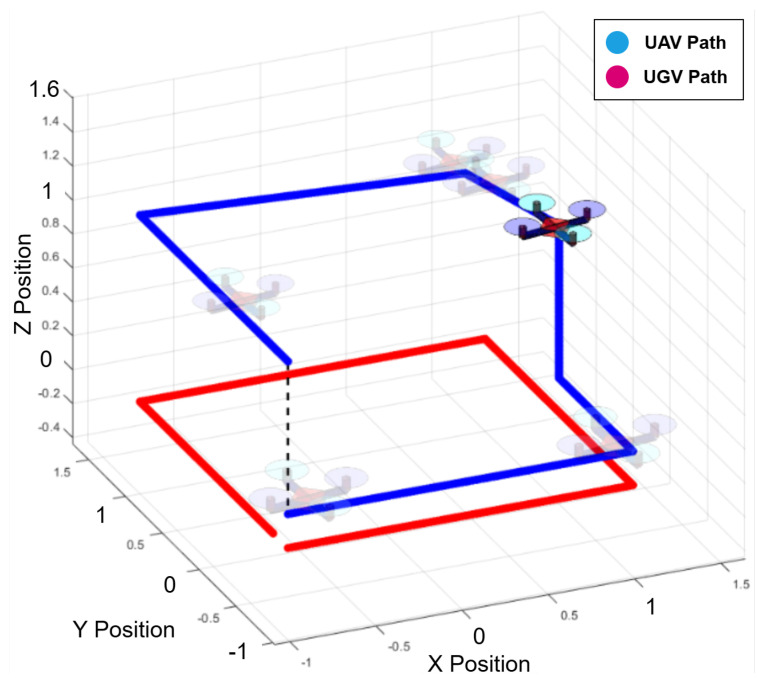
MATLAB-based trajectory visualization of the UAV–UGV inspection scenario. The UGV’s predefined path and the UAV’s inspection movements are shown to demonstrate collaborative coverage and spatial coordination.

**Figure 26:**
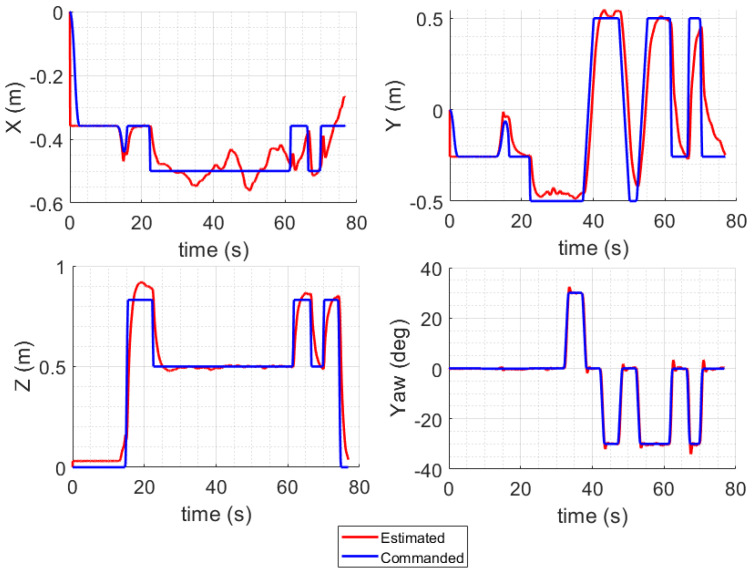
UAV tracking performance visualization from MATLAB during collaborative inspection. The QDrone 2’s real-time position is compared with its desired trajectory to assess control accuracy and flight stability.

As shown in Figure 25 and Figure 26, the collaborative inspection workflow between the UGV and UAV is demonstrated through their respective trajectories. The UGV followed a predefined path while the UAV maneuvered above to collect inspection data. The UAV leveraged YOLO for object detection and OCR to accurately read digital meters, with the UGV acting as a stable mobile base to support the inspection process.

### 6.2. UAV Flight Control and Real-Time Performance

The integration of the OptiTrack motion capture system ensured precise real-time localization and trajectory tracking, enabling effective synchronization between the two platforms. Additionally, HIL testing validated the UAV’s flight control system in real time, addressing potential issues before deployment in the real world. The system successfully facilitated data exchange between the UGV and UAV, ensuring smooth performance in the inspection scenario.

**Figure 27:**
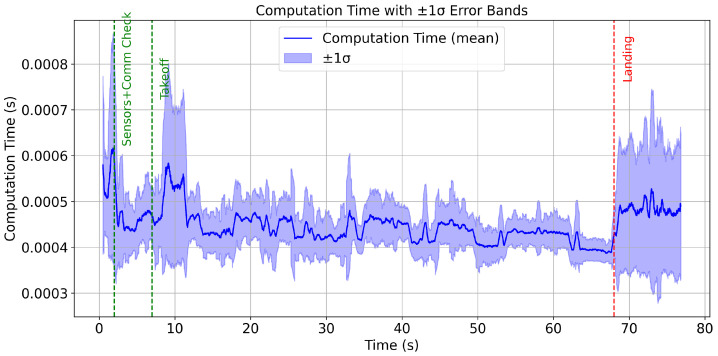
Computation time versus sample time analysis of the Simulink UAV control model during inspection. The figure illustrates system responsiveness and scheduling consistency under real-time processing constraints.

The inclusion of computation time and sample time plots provides valuable insights into the real-time performance of the QDrone 2 UAV during inspection tasks. These metrics are crucial for assessing the system’s responsiveness and efficiency under operational conditions, particularly in dynamic environments where rapid decision-making is imperative. The computation time plot illustrated in Figure 27 reflects the time taken by the UAV’s onboard processor to execute various control and sensing algorithms. Variability in computation time is expected, especially during complex tasks such as vision-based navigation or real-time obstacle avoidance. The sample time plot indicates the interval at which sensor data are sampled and processed. Consistent sampling intervals are crucial for maintaining the accuracy of navigation and control algorithms. Any irregularities in sample time, such as excessive delays, could degrade the system’s ability to respond to changes in the environment. The analysis of these plots underscores the importance of optimizing onboard computation and ensuring efficient utilization of system resources.

### 6.3. Vision-Based UAV Landing Accuracy

**Figure 28:**
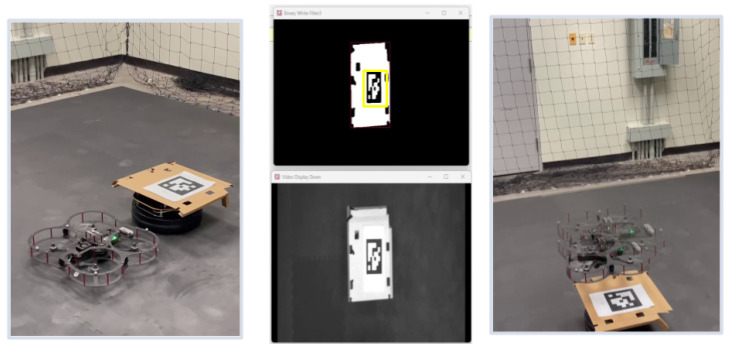
Sequential visualization of vision-based UAV landing using AprilTags. From left to right: UAV hovering above landing zone, grayscale camera feed, detected AprilTag with bounding box, and final descent onto the landing platform.

**Figure 29:**
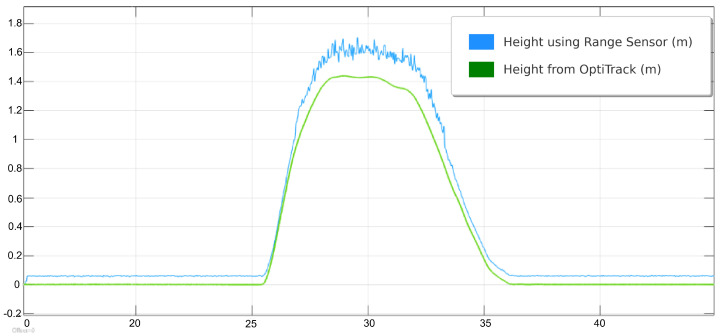
Height estimation comparison between the UAV’s onboard range sensor and the OptiTrack ground-truth system. The figure highlights discrepancies in sensor measurements during descent and emphasizes the importance of reliable altitude data fusion.

The average lateral position error during the landing process exceeded acceptable margins, with deviations of up to ±10 cm in some trials. This level of inaccuracy highlights the limitations of the real-time vision-based pose estimation approach under our experimental conditions, as shown in Figure 28. The UAV struggled to maintain consistent alignment with the AprilTag before descent, especially in scenarios where wind disturbances were present. The system operated at a frame rate of 20–25 FPS, which was lower than expected. This led to delays in processing the visual data and caused intermittent overshoots or undershoots in the UAV’s trajectory corrections. Figure 29 compares the height measurements obtained from different sensors: OptiTrack, IMU, and a range sensor. The OptiTrack data, represented by a smooth line, serves as the ground truth. In contrast, the height measurements from the IMU and range sensor exhibit some jitter, indicating potential noise or inaccuracies in these sensors. This comparison underscores the significance of sensor selection and data fusion techniques in achieving accurate height estimation in UAV applications.

### 6.4. Digital Meter Reading via AI and OCR

**Figure 30:**
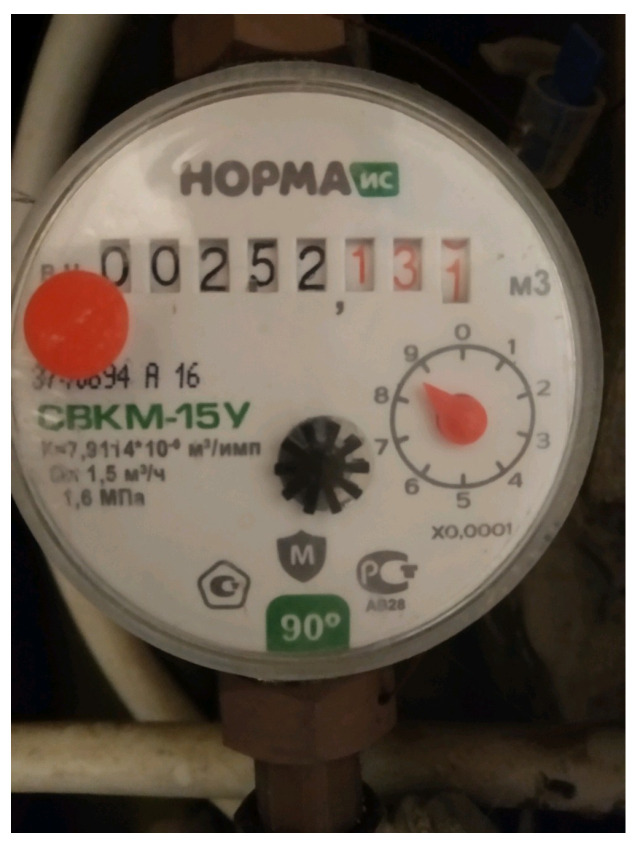
Sample digital meter gauge image captured by the UAV during inspection. The numerical display shown serves as the region of interest for segmentation and OCR-based reading.

**Figure 31:**
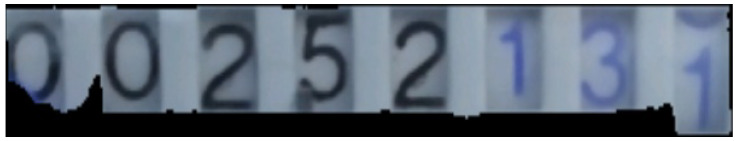
Example output from the trained U-Net model used for region-of-interest extraction. The figure shows successful segmentation of the meter display area from the original UAV-captured image.

The UAV was also utilized to capture digital meter gauge readings in a test environment. The captured images, which included both the digital meter gauge readings and the surrounding background, were sent to an end-user machine for a computer vision task. This task involved accurately extracting the meter gauge readings from the images and converting them into usable data. To achieve this, the first step was to isolate the area containing the digital numbers by masking the region of interest. To perform this masking, an AI model based on the U-Net segmentation architecture was developed and fine-tuned using our dataset, which comprised different meter gauge readings. During the training phase, the model was provided with original images and their corresponding masked versions. After training, the model could predict masks for unseen images when given only the original input. Figure 30 illustrates an example of an original image, while Figure 31 shows the output of the trained model, demonstrating its ability to generate a mask from an unseen input image.

**Figure 32:**
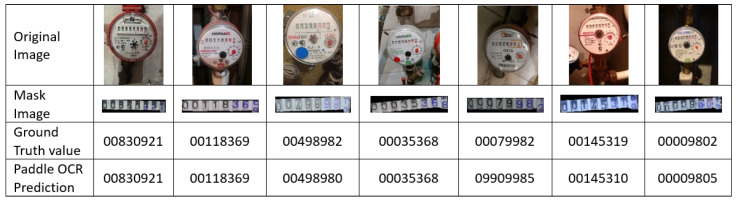
OCR results for masked digital meter images using PaddleOCR. The original image, segmented mask, ground-truth value, and predicted output are shown for seven representative samples. The system achieved 87.5% reading accuracy over 56 images.

To perform OCR, the masked images were processed using the Paddle OCR algorithm. The output of this algorithm was numerical values corresponding to the digital meter gauge readings. Figure 32 illustrates the original image, the masked image highlighting the region of interest, and the numerical predictions of the Paddle OCR algorithm for an 8-digit meter gauge reading. The second row of the table in Figure 32 shows the masked image produced by the trained model. The Ground-Truth Value row represents the actual meter gauge readings as manually recorded by a human. The last row displays the numerical output obtained by processing the masked image through the Paddle OCR algorithm. While the overall experimental dataset comprised 56 m gauge reading images, seven representative examples were selected for Figure 32 to illustrate the output. The Mask Image Model consistently demonstrated high precision in identifying the region of interest across all seven images. These masked images were then fed into the Paddle OCR model for value extraction. The OCR model showed varying levels of accuracy in extracting values, successfully identifying 49 out of 56 images, with seven images extracted incorrectly.

The accuracy of the Paddle OCR model on digital meter reading was calculated using the following formula:

(1) $$Accuracy=\left(\frac{N_{correct}}{N_{total}}\right)\times 100$$

where:

- $N_{correct}$ = Number of images with correctly extracted digits.
- $N_{total}$ = Number of total images.

(2) $$Accuracy=\frac{49}{56}\times 100=87.5\%$$

**Figure 33:**
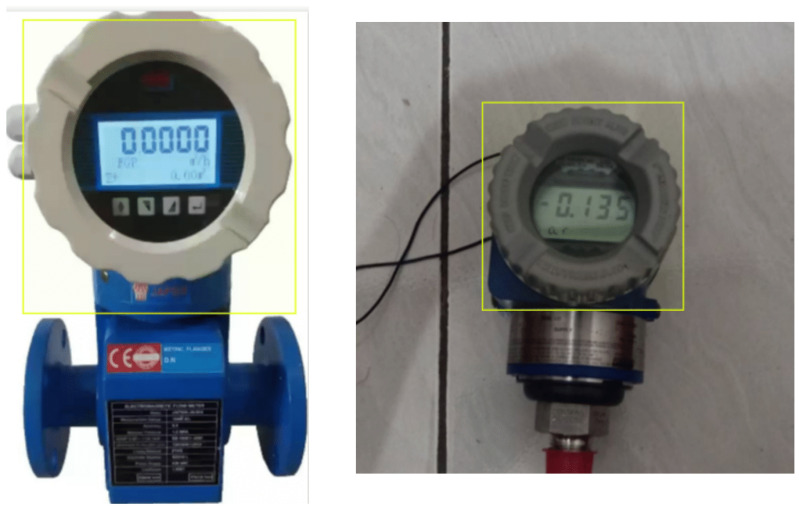
YOLOv5-based detection of digital meter gauges from UAV-captured images. The figure illustrates successful bounding box localization and object classification within the inspection scene.

**Figure 34:**
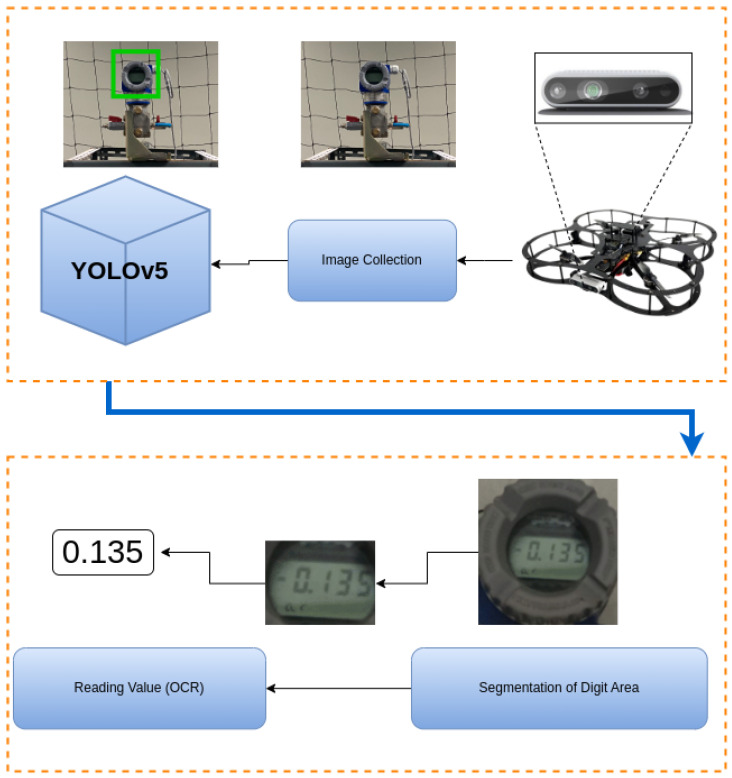
End-to-end system flowchart showing the pipeline from image acquisition and pre-processing to AI model training, inference, and OCR-based digital meter reading. This diagram summarizes the complete automation loop for the proposed UAV–UGV system.

For the tested dataset, Paddle OCR achieved an accuracy of 87.5% (49 correct extractions out of 56 total digits). Misclassifications were primarily due to the use of low-resolution images. Example detections using YOLO are shown in Figure 33 while the diagram in Figure 34 illustrates the process flow from image capture to the reading of digital meter gauge values using OCR. This figure provides a high-level view of the key stages involved in automating the meter gauge reading process. A total of 250 images were captured in varying real-world conditions, including different distances, angles, and lighting. These images were augmented to 560 through techniques such as brightness adjustment, reduced illumination, blurring, and rotation. The images were labeled using the CVAT AI program, with a focus on digital meters.

### 6.5. YOLO-Based Object Detection Performance

The dataset was split into training and validation sets (80:20 ratio) and used to train and validate the YOLO model. The performance of the model was evaluated using precision, recall, and Mean Average Precision (mAP) with example output shown in Figure 34. Precision indicates the proportion of correct predictions among all predicted samples, while recall measures the proportion of correctly identified digital meters. Since precision and recall often trade off, mAP provides a balanced evaluation of model performance, combining both accuracy and recall. Table 4 presents the performance metrics of the YOLOv5 object detection model. Precision, at 0.95, indicates a low rate of false positives, suggesting high accuracy in correctly identifying objects. A recall of 0.85 suggests that the model successfully detects 85% of the objects in the image. mAP (0–50) and mAP (50–95) measure the average precision at different IoU thresholds, with lower mAP (50–95) potentially indicating challenges in localizing objects with lower IoU scores. Overall, the model exhibits strong performance with high precision and recall, although further analysis is needed to address the potential localization challenges indicated by the mAP (50–95) value.

## 7. Conclusions, Limitations, and Future Work

This research examined the challenges associated with coordinating UAVs and UGVs, with an emphasis on efficient data exchange and optimized map representations to support robust inspection tasks. The study demonstrated that vision-based systems enhance inspection accuracy and reliability, particularly in scenarios that require high precision and adaptability to dynamic industrial conditions. Experimental validation of the navigation and control algorithms, as well as the vision intelligence features, confirmed the additional capabilities enabled by robotic automation and multi-domain collaboration.

One of the core contributions of this study is the development and validation of a SLAM framework for mapping and navigation for the UGV platform. The system integrates a two-dimensional LiDAR sensor and the SLAM Toolbox within the ROS environment. The TurtleBot 2 autonomously constructs accurate occupancy grid maps and navigates cluttered inspection scenarios. The framework was evaluated in both Gazebo simulation and a physical laboratory environment. Results demonstrate consistent localization, minimal pose drift, and reliable obstacle avoidance. The UGV’s mapping capabilities enable precise positioning and coordination with the UAV during joint inspection tasks, forming a foundational layer for future multi-agent robotic systems. These results confirm the approach’s suitability for real-time navigation in complex, GPS-denied industrial environments.

Although we have discussed multiple techniques for the integration of UGV and UAV systems, there are also limitations associated with this study. The scope of this study is to facilitate the integration of UGVs and UAVs within a laboratory environment. First, the current vision-based landing system performs reliably in controlled indoor conditions, but it remains sensitive to lighting, marker visibility, and disturbances such as wind. Future efforts will explore adaptive vision models, dynamic thresholding, markerless detection, and multimodal sensor fusion (e.g., integrating vision with IMU or range sensors) to improve stability under varied conditions and compare the results to alternative architectures. In a subsequent stage of a future study, we plan to perform SLAM benchmarking, evaluate different architectures, and compare the results with ground truth. Second, although the PaddleOCR pipeline achieved 87.5% accuracy in the lab conditions, future work will focus on expanding the dataset, especially with more challenging lighting and occlusion scenarios. We also aim to explore higher-resolution camera modules and optimized image-processing pipelines, such as utilizing the latest YOLO models, to reduce latency and enhance recognition accuracy during flight. We also plan to report distance and angle analyses, compare Paddle with other techniques, and conduct ablation studies. Lastly, the current system has been validated in a controlled lab setting. Due to this, we did not report timing diagrams from system components involving UGV, GCS, and UAV; therefore, we did not report latencies, jitter, and other performance metrics. Next steps include adapting the UAV–UGV coordination framework to handle outdoor or semi-structured environments, integrating fault-tolerant safety features, and optimizing SLAM and communication modules for multi-agent scalability and low-bandwidth operation. Additionally, we will compute end-to-end metrics, including timing diagrams.

## Figures and Tables

**Table 1:** Overview of Vision-Based UAV Navigation: Advantages, Challenges, and Use Cases.

Advantages	Challenges	Field of Application
Comprehensive visual data. Anti-jamming capabilities. Real-time data processing. High spatial accuracy. Reliable inspection.	Complex environments. Affected by adverse weather and lighting. Vulnerable to optical illusions. Requires real-time processing power.	Industrial inspection. Agriculture. Surveillance and monitoring. Security.

**Table 2:** Summary of Studies on SLAM and Automation Technologies.

Author(s)	Focus of the Study	Key Technologies/Approaches	Application/Outcome
Gao et al. (2024)	Distributed simulation architecture for heterogeneous robot collaboration	Three-layer simulation architecture	Facilitated multi-agent coordination for collaborative tasks
Sobczak et al. (2021)	Optimizing hardware configurations for SLAM tasks	Google Cartographer SLAM, LiDAR, IMUs, odometry	Improved decontamination processes through multi-sensor integration
Wu et al. (2021)	Automatic pointer meter gauge reading in low-light environments	Vision algorithms	Enhanced precision for meter gauge reading in substations
Hong et al. (2021)	Automatic water meter gauge reading in challenging environments	Image-based processing	Reliable meter gauge reading under diverse conditions
Li et al. (2020)	Robust automatic pointer meter gauge reading	Text detection algorithms	Increased reading accuracy in high-voltage environments
Rizk et al. (2019)	Survey of cooperative heterogeneous MRS	Task decomposition, coalition formation, task allocation	Framework for designing multi-robot systems to handle complex tasks
Yara Rizk et al. (2019)	Automation of complex tasks using heterogeneous MRS	Task complexity handling through coordination strategies	Defined cooperation levels for loosely/tightly coupled tasks
Chen et al. (2018)	SLAM-based indoor mapping in complex environments	LiDAR, depth cameras	High-accuracy mapping in corridors and libraries
Fang et al. (2013)	Meter gauge recognition for inspection robots	Robotic meter gauge recognition algorithm	Safer and more efficient inspection in industrial settings
Nguyen et al. (2010)	Autonomous control and SLAM in unmanned ground vehicles	SLAM algorithms, autonomous navigation	Enhanced simultaneous localization and mapping for UGVs

**Table 3:** Comparison of DJI Mavic 2 Pro and Quanser QDrone 2.

Feature	DJI Mavic 2 Pro	Quanser QDrone 2
Image	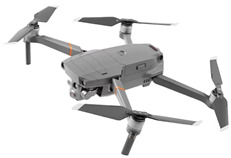	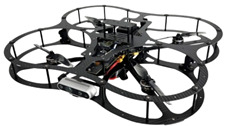
Primary Focus	Consumer-grade, high-end photography/videography	Research and Education, control systems development
Payload Capacity	Higher (e.g., can carry larger cameras)	Lower, optimized for control experiments
Flight Time	Generally longer	May have shorter flight times depending on payload and flight conditions
Camera Quality	Excellent image and video quality, advanced camera features	Varies depending on the camera module used, default is Intel RealSense D435 RGBD camera 30 FPS at 1080p
Software/SDK	Proprietary DJI software, limited access to low-level control	Comprehensive SDK and development tools (e.g., Quanser Quarc) for research and control algorithm development
Research Suitability	Limited for advanced control algorithms and research due to closed-source nature and limited access to low-level control	Highly suitable for research due to open-source nature, access to low-level control, and availability of development tools

**Table 4:** Result of YOLOv5 Training Performance.

Evaluation Metric	YOLOv5
Precision	0.95
Recall	0.85
mAP (0–50)	0.85
mAP (50–95)	0.5
Accuracy	0.84

## Data Availability

The data presented in this study are available on request from the corresponding author.
